# Testing the Limits of 454 Pyrotag Sequencing: Reproducibility, Quantitative Assessment and Comparison to T-RFLP Fingerprinting of Aquifer Microbes

**DOI:** 10.1371/journal.pone.0040467

**Published:** 2012-07-12

**Authors:** Giovanni Pilloni, Michael S. Granitsiotis, Marion Engel, Tillmann Lueders

**Affiliations:** 1 Institute of Groundwater Ecology, Helmholtz Zentrum München - German Research Centre for Environmental Health, Neuherberg, Germany; 2 Research Unit Environmental Genomics, Helmholtz Zentrum München - German Research Centre for Environmental Health, Neuherberg, Germany; Uppsala University, Sweden

## Abstract

The characterization of microbial community structure via 16S rRNA gene profiling has been greatly advanced in recent years by the introduction of amplicon pyrosequencing. The possibility of barcoding gives the opportunity to massively screen multiple samples from environmental or clinical sources for community details. However, an on-going debate questions the reproducibility and semi-quantitative rigour of pyrotag sequencing, similar to the early days of community fingerprinting. In this study we demonstrate the reproducibility of bacterial 454 pyrotag sequencing over biological and technical replicates of aquifer sediment bacterial communities. Moreover, we explore the potential of recovering specific template ratios via quantitatively defined template spiking to environmental DNA. We sequenced pyrotag libraries of triplicate sediment samples taken in annual sampling campaigns at a tar oil contaminated aquifer in Düsseldorf, Germany. The abundance of dominating lineages was highly reproducible with a maximal standard deviation of ∼4% read abundance across biological, and ∼2% across technical replicates. Our workflow also allows for the linking of read abundances within defined assembled pyrotag contigs to that of specific ‘*in vivo*’ fingerprinting signatures. Thus we demonstrate that both terminal restriction fragment length polymorphism (T-RFLP) analysis and pyrotag sequencing are capable of recovering highly comparable community structure. Overall diversity was roughly double in amplicon sequencing. Pyrotag libraries were also capable of linearly recovering increasing ratios (up to 20%) of 16S rRNA gene amendments from a pure culture of *Aliivibrio fisheri* spiked to sediment DNA. Our study demonstrates that 454 pyrotag sequencing is a robust and reproducible method, capable of reliably recovering template abundances and overall community structure within natural microbial communities.

## Introduction

Since the introduction of next generation sequencing technologies, a rapid growth of applications and developments in the screening of complex microbial communities has been observed [1], [2]. In particular, the characterization of bacterial 16S rRNA gene pools via amplicon pyrosequencing is becoming a method of choice and is currently overtaking previously used sequencing, and potentially even fingerprinting techniques. In fact, increasing numbers, quality and length of reads per run, together with the possibility of “barcode-tagging” sequences with sample-specific adaptors [3], gives the opportunity to massively screen multiple samples from various sources, and the potential to merge information on microbial phylogeny in samples with relative taxon abundance.

However, the general reproducibility and robustness of pyrotag sequencing, its potential to adequately recover relative template abundances, as well as its comparability to other, more established microbial community screening techniques like rRNA gene fingerprinting are still a matter of debate. The respective literature is continuously growing and provides both supportive [4], [5] and detrimental arguments [6], [7]. Besides generally accepted quality-filtering needs to avoid overestimation of diversity [7], reports on the technical reproducibility and semi-quantitative potential of pyrotag sequencing seem discouraging [6]. Recently, bias inherent to specific multiplexing identifier (MID) adaptors for barcode-tagging has also been reported [8], as well as systematic errors generated by the entire PCR, sequencing and data evaluation workflow [9]. This suggests that PCR-related errors may potentially be more difficult to eliminate than sequencing ones. On the other hand, studies on bacterial diversity in mangrove microcosms [5] and arctic tundra [4] reported robust reproducibility across Illumina and 454 sequencing replicates, respectively. Furthermore, in a recent clinical study on myelomonocytic leukemia [10], a high robustness and reproducibility of amplicon pyrosequencing was demonstrated across ten different laboratories in eight countries.

In our lab, we have recently established a bidirectional pyrotag sequencing strategy for bacterial 16S rRNA gene fragments, which allows not only for the phylogenetic affiliation of reads, but also assembly of dominating sequences and thus linking of pyrotag contigs to T-RFLP fingerprints of the same samples [11]. Here, we systematically investigate the reproducibility of this pyrotag sequencing approach across biological and technical replicates, we compare the recovery of relative operational taxonomic unit (OTU) abundances between pyrotag libraries and T-RFLP and, by spiking of environmental DNA extracts with qPCR defined template amendments, we explore the potential of the method for recovering input ratios of specific taxa within complex microbial communities.

## Materials and Methods

### Sample Acquisition and DNA Extraction

Environmental DNA was obtained from 9 sediment samples, three biological replicates each of one specific sediment depth sampled over three annual sampling campaigns at a tar oil contaminated aquifer in Düsseldorf, Germany [12]. Samples were collected via direct push drilling and shock-freezing of intact sediment liners on site. DNA was extracted from ∼0.8 g sediment aliquots as described [13] in triplicates (a, b and c) from samples taken at a similar depth (6.90 m in 2006, 6.67 m in 2008 and 6.85 m in 2009.

For DNA extraction, sediment aliquots were suspended in 650 µL PTN buffer (120 mM Na_2_HPO_4_, 125 mM Tris, 0.25 mM NaCl [pH 8]) and incubated at 37°C for 15 min with 40 µl lysozyme (50 mg ml^−1^) and 10 µl proteinase K (10 mg ml^−1^). After the addition of 150 µl 20% (wt/v) sodium dodecyl sulfate, incubation was continued for 15 min at 65°C with shaking at 500 rpm. The sediments were bead beaten (45 s at 6.5 ms^−1^ in a FastPrep-24 (MP Biomedicals, Solon, OH) with ∼0.2 ml of zirconia-silica beads (1∶1 mix of 0.1- and 0.7-mm diameter; Roth, Karlsruhe, Germany) and 100 µl of phenol-chloroform-isoamyl alcohol (25∶24:1) in 2-ml screw-cap vials. Afterwards nucleic acids were sequentially purified by extraction with 1 volume of phenol-chloroform-isoamyl alcohol (25∶24:1) and 1 volume of chloroform-isoamyl alcohol (24∶1). Purified nucleic acid was then precipitated with 2 volumes of 30% polyethylene glycol by incubation at 4°C for at least 2 h and subsequently centrifuged at 20,000 *g* and 20°C for at least 30 min. All used chemicals were from Sigma-Aldrich, St. Louis, MO, if not otherwise stated. For each biological sample, two parallel sediment extractions were pooled in 60 µl of elution buffer and stored at −20°C until further analysis.

### Pyrotag Library Preparation

Amplicon pyrosequencing was performed on a 454 GS FLX Titanium system (Roche, Penzberg, Germany) as recently described [11]. Briefly, barcoded amplicons for multiplexing were prepared using the primers Ba27f (5′-aga gtt tga tcm tgg ctc ag-3′) and Ba519r (5′-tat tac cgc ggc kgc tg-3′) extended with the respective A or B adapters, key sequence and multiplex identifiers (MID) as recommended by Roche. Pyrotag PCR was performed in a Mastercycler ep gradient (Eppendorf, Hamburg, Germany) with the following cycling conditions: initial denaturation (94°C, 5 min), followed by 28 cycles of denaturation (94°C, 30 s), annealing (52°C, 30 s) and elongation (70°C, 60 s). Each 50 µl PCR reaction contained 1× PCR buffer, 1.5 mM MgCl_2_, 0.1 mM dNTPs, 1.25 U recombinant Taq polymerase (Fermentas, St. Leon-Rot, Germany), 0.2 µg µl^−1^ bovine serum albumin (Roche), 0.3 µM of each MID-primer (Biomers, Ulm, Germany) and 1 µl of template DNA. Amplicons were purified using Agencourt AMPure-XP beads (Beckman Coulter, Brea, CA) and pooled in an equimolar ratio of 10^9^ molecules µl^−1^ as quantified by the Quant-iT PicoGreen dsDNA quantification kit (Invitrogen, Paisley, UK). Emulsion PCR, emulsion breaking and sequencing were performed applying the GS FLX Titanium chemistry following supplier protocols. Further details on amplicon mixes, sequencing and trimming statistics are provided in Supplementary Table S1. One library was prepared for each sediment DNA (biological replicates). Two additional libraries were prepared for one selected DNA extract (DNA replicate c of 2006), thus representing three technical replicates.

### T-RFLP Fingerprinting

T-RFLP fingerprinting of all above DNA templates was performed as described [11], using the same forward primer (FAM-labelled) as for pyrotag sequencing (Ba27f) and a distinct reverse primer (907r). This facilitates the linking of observed T-RFs to restriction sites predicted for assembled pyrotag contigs [11]. The very limited selection of replicate sediment samples presented in this technical report is part of a more comprehensive time- and depth-resolved study (38 replicate sediment samples in total, to be reported elsewhere), fully analysed by T-RFLP and to a large extent also by pyrotag sequencing.

### Spiking Experiment

For defined template amendments, we quantified bacterial rRNA gene copy numbers in one environmental DNA extract (replicate c, 2006) via quantitative PCR as described elsewhere [14]. The same was done for a genomic DNA extract of *Aliivibrio fisheri* (strain NRRL B-11177; provided by Aboatox Oy, Masku, Finland), a marine bacterium not expected to be detectable in the original aquifer sample. In the following, defined amounts (0.2, 2 and 20%) of quantified *A. fisheri* rRNA genes (genomes, respectively) were spiked (triplicate series of mixtures) to the sediment DNA. Amplicon pyrosequencing was performed for two of these series (duplicates) as described above. Moreover, the third replicate series of template mixtures was sequenced after a 2-step PCR for pyrotag generation, to address possible biases introduced by barcoded primers in 1 vs. 2-step pyrotag amplification as recently reported [8]. For the latter, 28 PCR cycles with non-MID-tagged bacterial primers (Ba27f/Ba519r) were followed by 5 cycles with MID-primers, using 2 µl of the first PCR products as template for the second round of amplification. Further details on the sequencing of these amplicon pools are provided in Supplementary Table S2.

### Pyrotag Data Handling

Quality filtering of the pyrosequencing reads was performed using the automatic amplicon pipeline of the GS Run Processor (Roche) with a modification of the valley filter (vfScanAll- Flows false instead of TiOnly) to extract sequences. Afterwards, reads were further quality-trimmed using the TRIM function of GREENGENES [15] with default settings. Reads shorter than 250 bp (after trimming) and with incorrect sequencing primers were excluded from further analysis. Read affiliation was done for combined forward and reverse reads for each library using the RDP classifier [16] with confidence threshold set to 80% (default). The RDP pyrosequencing pipeline [17] was also used to generate Shannon diversity indices *H’* and for overall community comparison based on linkage clustering of detected OTUs (97% sequence similarity). Sørensen indices of similarity [6] were calculated across biological and technical replicates.

Contigs for T-RF prediction of dominating amplicons were assembled with SEQMAN II software (DNAStar, Madison, WI), using forward- and reverse-reads, as described [11]. Thresholds of read assembly into one contig were set to at least 98% sequence similarity for a minimum overlap of 50 bp. Contigs within one library with less than 20 reads and not at least one forward and one reverse read were excluded from further analysis. *In silico* T-RF prediction was performed using TRiFLe [18], and predicted T-RFs were correlated to the numbers of reads within the most abundant contigs. For comparison of overall community structure between T-RFLP fingerprints generated *in vivo* and *in silico* fingerprints inferred from assembled contigs, the functional organisation (*Fo*) of communities was inferred from Pareto-Lorentz curves [19]. Here, OTU abundance is summarised from abundance-ranked OTUs, and the cumulative abundance of the most abundant 20% of OTUs is recorded. *Fo* was inferred from all T-RFs of a given fingerprint passing the threshold of 100 relative fluorescence units in electrophoresis [13], or from all contigs of a given library containing 20 or more reads after assembly. A *Fo* of 25, 45 and 80% refers to a low, medium or high functional organisation of the community, respectively [19], and is an inverse measure of community evenness.

All raw and trimmed reads generated in this study have been deposited in NCBI’s Gene Expression Omnibus [20] and are accessible through GEO series accession number GSE35631, except for sequences from the biological replicates of group a, which were previously deposited under the GEO series GSE25345 (accession numbers: GSM623520, GSM623524 and GSM623529).

## Results and Discussion

**Figure 1:**
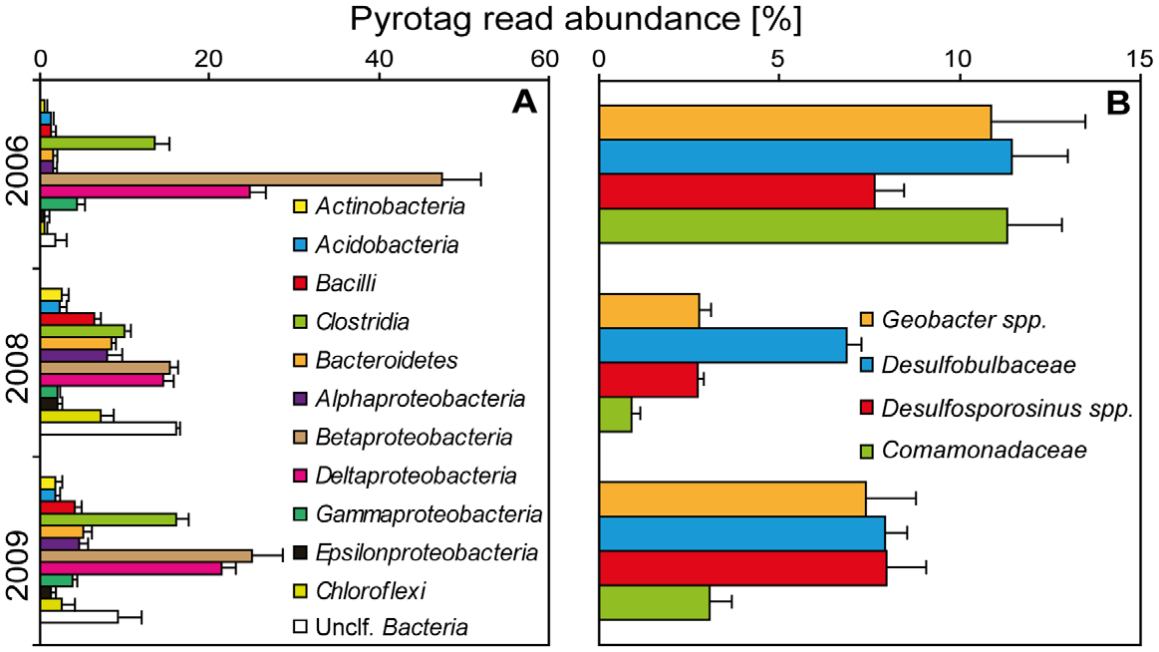
Reproducibility of pyrotag read abundance over biological replicates of aquifer DNA extracts. Error bars indicate standard deviation (positive only) of averaged triplicate samples for each year. Taxon classification was recorded at phylum/class level (A) for entire libraries, and for selected abundant taxa (B) detected at the site [11], [13].

**Figure 2:**
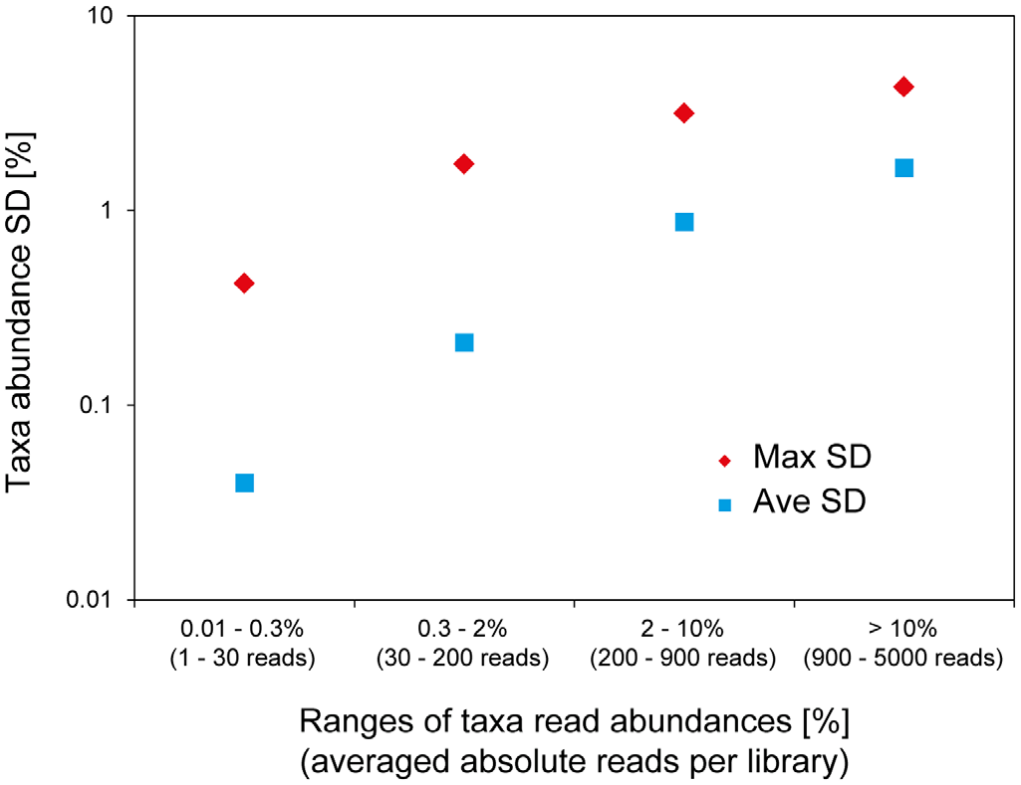
Read abundance standard deviation (SD) for dominating and less abundant taxa in pyrotag libraries. Averaged and maximal SDs for taxa within specified abundance ranges are given. All taxa resolved by the RDP-classifier and recovered at 1 read in average or more over 9 sediment pyrotag libraries were included in the calculation.

The reproducibility of pyrotag sequencing in our workflow was generally high, with phylum/class-level read abundances across biological replicates retrieved at an average standard deviation (SD) of ∼0.6% (Figure 1A, maximum SD of 4.3%). Also at a higher, family- or genus-level resolution, selected dominating members of the aquifer microbial community were retrieved at comparable reproducibility (average SD of ∼0.9%, max. SD of 2.7%; Figure 1B). Over all different taxonomic levels and abundances of taxa defined by the RDP classifier, abundant ones (>10% abundance, typically phylum, class, or order-level resolution) were recovered with an average SD of ∼1.7% read abundance (Figure 2). Less abundant taxa were retrieved at comparable relative reproducibility, e.g. with an average SD of 0.9, 0.2 or 0.04% for taxa below either 10%, 2%, or 0.3% read abundance, respectively. Reproducibility was comparable, or even better for technical replicates, where phylum-level max. SD was 2% read abundance (supporting Figure S1).

Linkage clustering-based comparison of OTU occurrence (97% sequence similarity) showed that 45±5% of OTUs in each library were present as singletons (only one read per library). Nevertheless, OTUs overlap (Sørensen similarity) for triplicated pyrotag libraries was high, and at 88, 82, and 67% for sediments of 2006, 08 and 09, respectively. This is in contrast to a much lower OTU overlap reported recently for replicated pyrotag libraries from soil [6]. A number of different factors may have contributed to this distinction: the soil samples may have harboured a substantially more diverse bacterial community than our aquifer sediments, making effects of undersampling in pyrotag libraries more severe. Second, that study used post-PCR ligation of 454 sequencing adaptors and ‘classical’ 454 sequencing, while our amplification primers already contained sequencing adaptor tags and Titanium chemistry was used. The comparative effects of both on pyrotag library reproducibility have not been specifically addressed to date. Last but not least, our workflow integrates an initial quality trimming step of sequencing reads based on base calling confidence scores [15], the effects of which on the reduction of sequencing noise and overall library similarity should not be underestimated.

In our hands, total OTU overlap of pyrotag libraries from technical replicates was even higher (>96%). Thus to a certain extent, also singleton OTUs seemed reproducible over replicated libraries. We can cautiously speculate on whether such singletons already represent members of the rare biosphere, however much ‘deeper’ sequencing (i.e. >100.000 s, not ∼10.000 reads per library) would have been necessary to truly address this question. We expect that while ‘deeper’ sequencing would not have altered the reproducibility of abundant OTUs, overall diversity and also the stochastic appearance of rare OTUs would have increased. Also Shannon diversity *H’* was highly reproducible for biological replicates of pyrotag libraries and comparable to *H’* reproducibility obtained in T-RFLP fingerprinting of the same samples (Table 1). However, total T-RF diversity was only ∼50% of pyrotag OTU diversity, which was expected, since taxon-specific resolution of pyrotag sequencing is much higher than fingerprinting.

**Table 1:** Reproducibility and comparison of Shannon diversity (*H*’) and selected OTU abundance in T-RFLP fingerprinting and pyrotag libraries.

	2006		2008		2009	
	Aver.	± SD	Aver.	± SD	Aver.	± SD
**Diversity**						
T-RFLP *H’*	2.3	0.1	2.3	0.1	2.6	0.1
Pyrotag *H‘*	4.8	0.2	5.8	0.2	5.7	0.2
***Geobacter*** ** spp. abundance**						
129 bp T-RF	11.6	4.6	1.3	2.3	6.0	1.1
* Geobacter* spp. reads	10.8	2.6	2.8	0.3	7.4	1.4
***Desulfobulbaceae*** ** abundance**						
159 bp T-RF	15.2	1.1	8.4	1.3	15.5	1.4
* Desulfobulbaceae* reads	11.4	1.6	6.9	0.4	7.9	0.6
***Desulfosporosinus*** ** spp. abundance**						
228+137 bp T-RFs	12.5	3.0	2.1	0.2	9.8	1.6
* Desulfosporosinus* reads	7.6	0.8	2.7	0.2	8.0	1.1
***Comamonadaceae*** ** abundance**						
486 bp T-RF	8.8	1.9	0.0	0.0	1.7	0.1
* Comamonadaceae* reads	11.3	1.5	0.9	0.3	3.1	0.6

**Figure 3:**
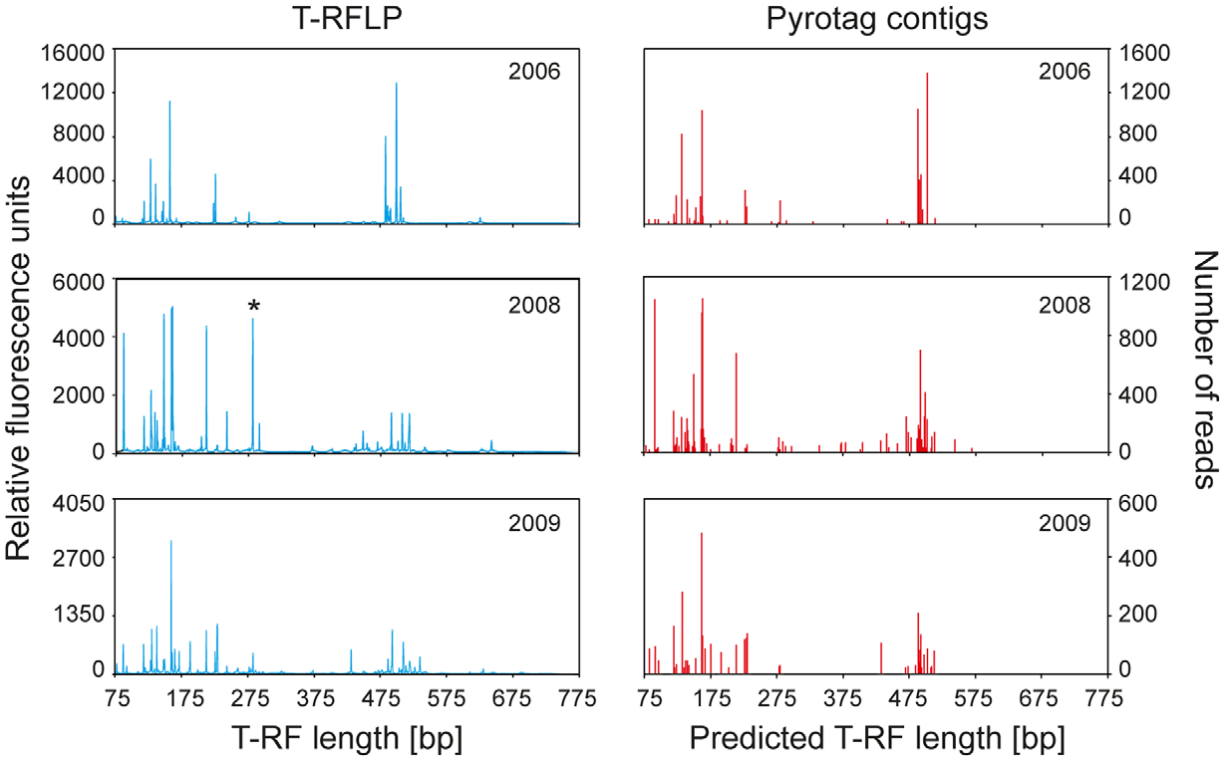
Comparison of bacterial community structure as recovered in T-RFLP fingerprinting and pyrotag libraries. Results are shown for selected aquifer sediment DNA extracts (replicate *b* from each year). For pyrotag ‘fingerprints’, the abundance of dominating assembled contigs is illustrated via T-RFs predicted *in silico* along with the total number of reads contributing to that contig. (*) illustrates the 282 bp pseudo T-RF of specific *Spirochaete* populations detected as *in vivo* T-RF only.

As our workflow allows for the linking of read abundances within defined assembled contigs to that of specific ‘*in vivo*’ T-RFs, we compared OTU abundance retrieved via both methods for representative samples (Table 1 and Figure 3). With this unique approach, it was possible to demonstrate the highly similar community structure and OTU abundance patterns retrieved by both methods. In essence, it becomes clear that both T-RFLP and pyrotag sequencing are capable of recovering the same amplicon pools from environmental samples, and yield highly comparable overall microbial community patterns. As expected, T-RFs predicted *in silico* vs. those measured *in vivo* mostly differed by a few bp [13], which prevented a straightforward calculation of overall community similarity e.g. via Sørensen OTU overlap or comparable indices. Nevertheless, the functional organisation (*Fo*) of bacterial communities as inferred from Pareto-Lorentz curves of cumulative OTU abundances [19] for both approaches supported highly similar overall community structure. Thus, *Fo* was 0.76 vs. 0.71 for in *vivo* T-RFs vs. pyrotag contigs in 2006, and 0.53 vs. 0.54 in 2009. In 2008 however, inferred *Fo* was higher for T-RFs than for pyrotag contigs (0.74 vs. 0.59), which can be explained by the exceptionally high yield of reads recovered in that specific pyrotag library (Table S1), resulting in more OTUs from assembled contigs passing our subjectively defined 20 reads-per-contig threshold. A library-specific cut-off for defining contig assembly read thresholds could help to alleviate this limitation of our present workflow.

The most significant difference in T-RF patterns of both methods was related to an abundant 282 bp T-RF found ‘*in vivo’* in the 2008 sample, but not predicted ‘*in silico*’ for any dominating pyrotag contig (Figure 3). Upon second consideration, this was identified as a pseudo T-RF [21] of abundant *Spirochaetes*-related templates in this sample, for which a secondary *Msp*I restriction site at 285 bp was predicted, succeeding their primary restriction site at 208 bp. Thus it is plausible that this population was actually represented by both the 204 and 282 bp T-RFs detected ‘*in vivo*’, with the second one being a pseudo T-RF. Other, minor differences were related to a higher relative abundance of specific ‘*in vivo*’ T-RFs compared to the respective pyrotag contigs. This was expected, since it is known that several phylogenetically distinct rRNA gene populations may share identical terminal restriction sites, and thus contribute to the same ‘*in vivo*’ T-RFs. In fact, especially the 159 bp T-RF, representing a population of primary toluene degraders (unclassified *Desulfobulbaceae*) *in situ* [11], was up to 2-fold more abundant in fingerprinting than in pyrotag sequencing (Table 1). Thus here, additional bacterial populations may have contributed to this T-RF. For other important populations at the site such as *Geobacter* spp., *Desulfosporosinus* spp. and the *Comamonadaceae* [11], [13], the abundance of the respective T-RFs (129 bp; 137 & 228 bp; 486 bp) was more similar to pyrotag read abundance. Nevertheless, also the fact that different reverse primers were used in both approaches (Ba519r for pyrotags vs. 907r for T-RFLP) may have introduced further distinctions in the recovery of community structure cannot be excluded.

Amplicon pyrosequencing seems to hold also the potential for a quantitative recovery of template input ratios. Spiked *A. fisheri* reads were retrieved with linearity (R^2^ = 0.99) over three orders of magnitude reflecting qPCR-defined amendment ratios in a reliable manner (Table 2). The maximum amendment (20%) of external *A. fisheri* DNA caused decreases in abundance of maximally ∼2% for other important intrinsic taxa (Figure S2). This linear representation is again in contradiction to the apparently random recovery of template amendment ratios recently reported [6]. In that study, 0.1% of genomic DNA of *S. oneidensis* was spiked to soil DNA. Very likely, the use of qPCR guided template spiking differentiate our results. It is clear that DNA with quantified 16S rRNA gene content can be spiked in a much more meaningful manner than amendments guided by bulk DNA quantification. qPCR-defined template mixtures have already been crucial earlier, in demonstrating the semi-quantitative robustness of rRNA gene-based T-RFLP fingerprinting [22]. Nevertheless, also the possibly pronounced impact of our initial pyrotag data quality control measures (confidence trimming) on template abundances, linear amendment recovery, and overall library similarity cannot be ignored.

**Table 2:** Semi-quantitative recovery of spiked *A. fisheri* 16S rRNA genes in pyrotag libraries.

*A. fisheri* amendment	*A. fischeri* pyrotag reads			
[%]	Aver. [reads]	± SD	Aver. [%]	± SD
20	1894	416	23.8	1.20
2.0	266	51	3.9	0.44
0.2	24	0.1	0.3	0.11
0.0	0	0.6	0.0	0.01

Finally, we want to mention that our comparison of 1- and 2-step PCR for the generation of pyrotag libraries did not produce pronounced distinctions in read abundance of dominating lineages and overall community structure (Figure S2). In accordance to a recent report [8], 2-step pyrotag libraries were significantly more diverse (Supplementary Table S2). In our hands, 2-step pyrotag libraries also contained ∼5 times higher ratios of shorter reads (<250 bp). We are still examining whether this phenomenon could potentially be connected to the apparently increased diversity of 2-step libraries. However, the 2-step PCR did not affect the semi-quantitative recovery of *A. fisheri* 16S rRNA gene sequences (Figure S2).

Since each template of our study was screened using different MID adaptors and also variable pools of amplicon preparation and mixing (Supplementary Table S1), the highly reproducible read abundances obtained across biological and technical replicates seem to suggest that none of the used MID adaptors introduced a systematic, adaptor specific bias in overall community structure, or that all were connected to a similar bias. Nevertheless, since we did not systematically compare 1-step vs. 2-step amplicon libraries for technical replicates generated with the same MID adaptors, these results do not exclude potential biases introduced by specific MID adaptors.

In conclusion, our study demonstrates that 454 pyrotag sequencing is a robust and reproducible method for the reliable recovery of the diversity and structure of complex natural microbial communities, with a reproducibility certainly comparable to that of established screening tools such as T-RFLP fingerprinting. As for every other analytical technique, biological and technical replication is essential to obtain an accurate measure of semi-quantitative results [23]. Nevertheless, each of our pyrotag libraries showed consistent read abundances with subsequent replicate means, and the most important community distinctions were recovered also in non-replicate libraries. The possibility of spiking quantitative template amendments to pyrotag libraries brings new exciting applications in the study of complex microbial communities. Amendment with multiple standards not present in the sample, or even with synthetic DNA, could be an interesting future development.

## Supporting Information

Figure S1 **Reproducibility of pyrotag read abundance across technical replicates of one aquifer sediment DNA extract.** Results from a representative extract (replicate c, 2006) are shown only for the most abundant phyla as detailed in Figure 1.
(TIF)

Figure S2 **Quantitative recovery and reproducibility of pyrotag read abundance for selected dominating taxa in spiking experiment.** Sediment DNA was spiked with defined amendments (20, 2, 0.2 and 0%) of *Aliivibrio fisheri* rRNA genes. Two series (0–20%) of amended sediment DNA (extract c, 2006) were analysed in technical duplicates via one-step PCR (A), and one series of amendments via two-step PCR (B).
(TIF)

Table S1 **Number of reads and average read lengths for pyrotag libraries of natural aquifer sediments.**
(DOC)

Table S2 **Number of reads and average read lengths for pyrotag libraries from spiking experiment.**
(DOC)
