# Supplementary figures and images for: Testing the Limits of 454 Pyrotag Sequencing: Reproducibility, Quantitative Assessment and Comparison to T-RFLP Fingerprinting of Aquifer Microbes
Source: PLoS One. 2012 Jul 12;7(7):e40467. doi: 10.1371/journal.pone.0040467 (PMC3395703; doi:10.1371/journal.pone.0040467)

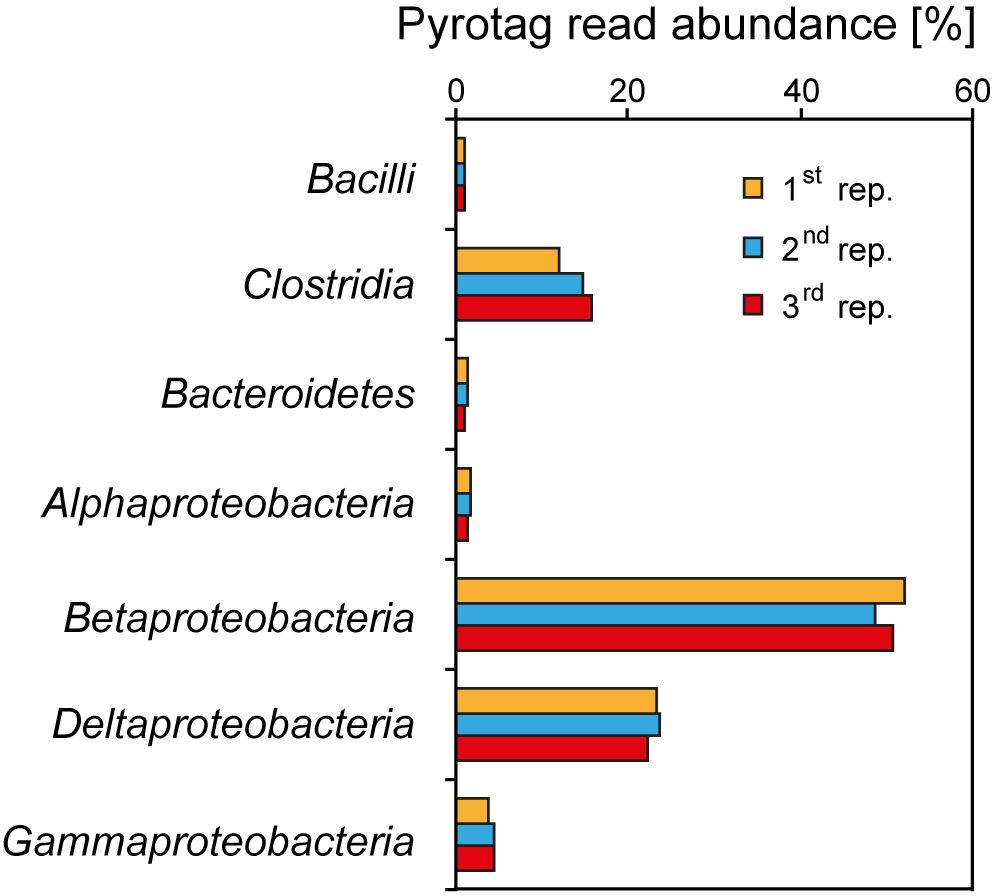

Supplement: Figure S1 — Reproducibility of pyrotag read abundance across technical replicates of one aquifer sediment DNA extract. Results from a representative extract (replicate c, 2006) are shown only for the most abundant phyla as detailed in Figure 1. (TIF) [file pone.0040467.s001.tif]

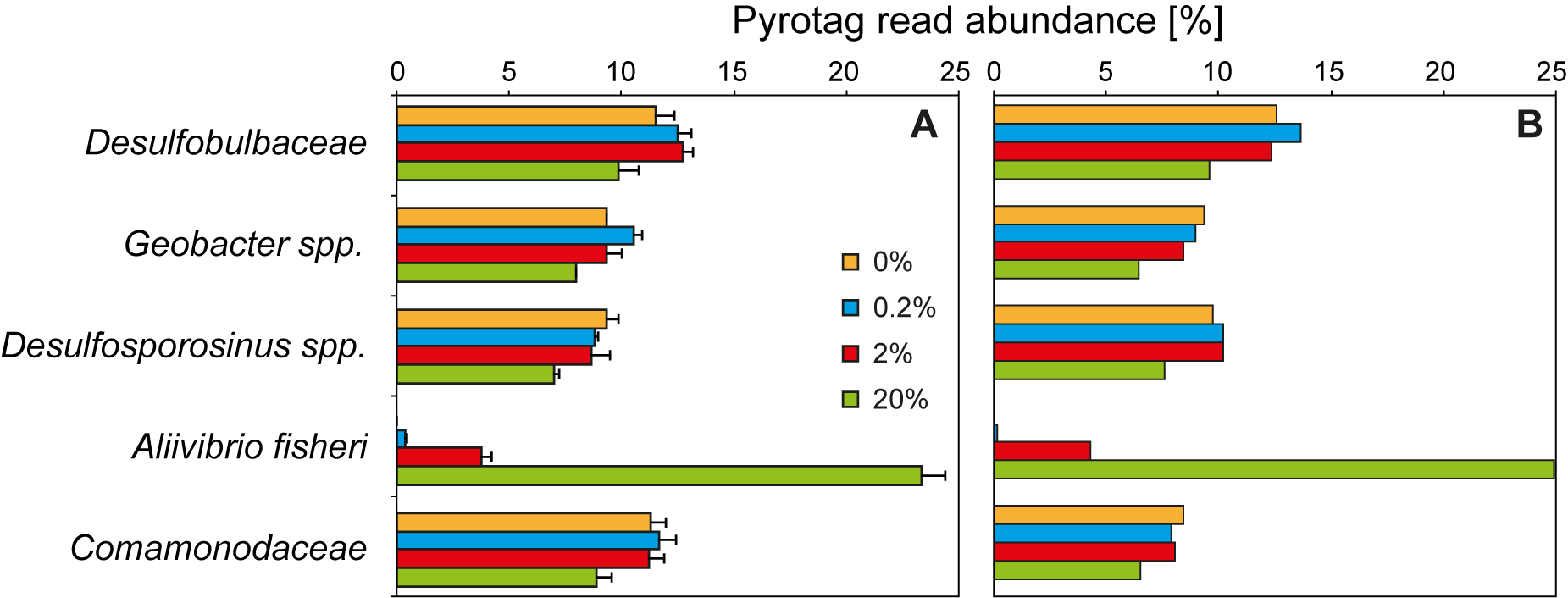

Supplement: Figure S2 — Quantitative recovery and reproducibility of pyrotag read abundance for selected dominating taxa in spiking experiment. Sediment DNA was spiked with defined amendments (20, 2, 0.2 and 0%) of Aliivibrio fisheri rRNA genes. Two series (0–20%) of amended sediment DNA (extract c, 2006) were analysed in technical duplicates via one-step PCR (A), and one series of amendments via two-step PCR (B). (TIF) [file pone.0040467.s002.tif]
