# Supplementary material for: Testing the Limits of 454 Pyrotag Sequencing: Reproducibility, Quantitative Assessment and Comparison to T-RFLP Fingerprinting of Aquifer Microbes
Source: PLoS One. 2012 Jul 12;7(7):e40467. doi: 10.1371/journal.pone.0040467 (PMC3395703; doi:10.1371/journal.pone.0040467)
Supplement: Table S1 — Number of reads and average read lengths for pyrotag libraries of natural aquifer sediments. (DOC) [file pone.0040467.s003.doc]

**Table S1.** Number of reads and average read lengths for pyrotag libraries of natural aquifer sediments.

|  | **2006** | | | **2008** | | | **2009** | | |
| --- | --- | --- | --- | --- | --- | --- | --- | --- | --- |
| **Replicate** | **a** | **b** | **c** | **a** | **b** | **c** | **a** | **b** | **c** |
| **DNA extraction [date]** | 12.10.09 | 02.11.09 | 30.11.09 | 28.01.10 | 02.02.10 | 05.02.10 | 01.09.09 | 08.09.09 | 02.11.09 |
| **Library run [date]** | 05.05.10 | 08.11.11 | 08.11.11 | 24.03.10 | 08.11.11 | 08.11.11 | 05.05.10 | 08.11.11 | 08.11.11 |
| **Fraction of picotitre plate** | 1/8 | 1/4 | 1/4 | 1/8 | 1/4 | 1/4 | 1/8 | 1/4 | 1/4 |
| **Total samples per pool** | 14 | 26 | 26 | 9 | 26 | 26 | 14 | 26 | 26 |
| **Total reads** | 6281 | 10362 | 12390 | 11438 | 16095 | 9087 | 7909 | 6116 | 9740 |
| **Forward reads** | 3116 | 2953 | 3691 | 3857 | 4869 | 2730 | 4097 | 1945 | 2963 |
| **Reverse reads** | 3165 | 7240 | 8532 | 7581 | 10743 | 6143 | 3812 | 4032 | 6515 |
| **Average length total reads [bp]** | 507 | 511 | 505 | 490 | 513 | 512 | 501 | 514 | 509 |
| **Quality trimmed (>250 bp) reads** | 5299 | 9492 | 11189 | 6055 | 14668 | 8369 | 4887 | 5654 | 8854 |
| **Forward reads, trimmed** | 2522 | 2657 | 3333 | 2772 | 4535 | 2556 | 2402 | 1816 | 2692 |
| **Reverse reads, trimmed** | 2777 | 6835 | 7856 | 6313 | 10133 | 5813 | 2485 | 3838 | 6162 |
| **Average length trimmed [bp]** | 360 | 429 | 432 | 365 | 430 | 428 | 359 | 428 | 429 |
| **Shannon Index (H')** | 4.7 | 4.3 | 4.6 | 5.9 | 5.5 | 5.4 | 5.8 | 5.4 | 5.3 |
