# Supplementary material for: Testing the Limits of 454 Pyrotag Sequencing: Reproducibility, Quantitative Assessment and Comparison to T-RFLP Fingerprinting of Aquifer Microbes
Source: PLoS One. 2012 Jul 12;7(7):e40467. doi: 10.1371/journal.pone.0040467 (PMC3395703; doi:10.1371/journal.pone.0040467)
Supplement: Table S2 — Number of reads and average read lengths for pyrotag libraries from spiking experiment. (DOC) [file pone.0040467.s004.doc]

**Table S2.** Number of reads and average read lengths for pyrotag libraries from spiking experiment^1^.

|  | **1-step PCR, 1^st^ rep.** | | | | **1-step PCR, 2^nd^ rep.** | | | | **2-step PCR, 3^rd^ rep.** | | | |
| --- | --- | --- | --- | --- | --- | --- | --- | --- | --- | --- | --- | --- |
| ***A. fischeri* amendment [%]** | **20** | **2** | **0.2** | **0** | **20** | **2** | **0.2** | **0** | **20** | **2** | **0.2** | **0** |
| **Total reads** | 7598 | 8227 | 9420 | 9716 | 10609 | 8258 | 8927 | 5519 | 9219 | 6471 | 7038 | 5628 |
| **Forward reads** | 3156 | 3268 | 3795 | 3987 | 4303 | 3244 | 3483 | 2150 | 3374 | 2587 | 2690 | 2181 |
| **Reverse reads** | 4345 | 4851 | 5485 | 5594 | 6158 | 4943 | 5309 | 3290 | 5676 | 3720 | 4253 | 3310 |
| **Average length total reads [bp]** | 517 | 512 | 512 | 512 | 509 | 512 | 513 | 516 | 424 | 452 | 453 | 467 |
| **Quality trimmed (>250 bp) reads** | 7229 | 7709 | 8885 | 9121 | 9918 | 7752 | 8395 | 5221 | 6756 | 5036 | 5480 | 4528 |
| **Forward reads, trimmed** | 3031 | 3086 | 3606 | 3765 | 4089 | 3059 | 3294 | 2028 | 2715 | 2170 | 2164 | 1828 |
| **Reverse reads, trimmed** | 4198 | 4623 | 5279 | 5356 | 5829 | 4693 | 5101 | 3193 | 4041 | 2866 | 3316 | 2700 |
| **Average length, trimmed [bp]** | 442 | 440 | 441 | 445 | 438 | 439 | 442 | 441 | 442 | 443 | 444 | 447 |
| **Shannon index (*H*')** | 3.7 | 4.3 | 4.1 | 4.2 | 4.2 | 4.3 | 4.2 | 3.9 | 4.7 | 4.7 | 4.7 | 4.6 |

^1^ Sediment replicate DNA extract *c* from 2006 was spiked with qPCR quantified amendments (20, 2, 0.2 and 0%) of rRNA genes of *Aliivibrio fisheri*. Independent triplicate series of spiking mixes were analysed twice via 1-step PCR and once via 2-step PCR. All 12 templates were loaded on 1/8^th^ of a FLX picotitre plate run in Nov. 2011.
